# Family-centered nursing interventions on asthma control and caregiver management capacity in preschool children with asthma: a systematic review and meta-analysis

**DOI:** 10.3389/fped.2026.1949534

**Published:** 2026-09-25

**Authors:** Yiling Pan, Xuefen Ye

**Affiliations:** 1 Department of Nursing, Hangzhou Children’s Hospital, Hangzhou, Zhejiang, China; 2 Department of Gastroenterology, Hangzhou Children’s Hospital, Hangzhou, Zhejiang, China

**Keywords:** asthma, children, disease management, family-centered nursing, meta-analysis, preschool children, randomized controlled trial, systematic review

## Abstract

**Introduction:**

Systematic evaluation of the impact of family-centered nursing interventions on asthma control and caregiver management abilities in preschool children with asthma.

**Methods:**

Randomized controlled trials (RCTs) published from database inception to December 2025 were searched in PubMed, Embase, Web of Science, CINAHL, Scopus, and Chinese databases. Literature screening and data extraction were independently completed by two researchers, and the quality was evaluated by the Cochrane risk-of-bias tool for randomized trials (RoB 2.0). Meta-analysis was performed using RevMan 5.4 software.

**Results:**

A total of 15 RCTs involving 457 children were included. The meta-analysis results showed that, compared with routine care, family-centered nursing interventions significantly improved the Asthma Control Test score (MD = 2.49, 95% CI: 1.15–3.83), quality of life score (SMD = 1.02, 95% CI: 0.52–1.51), and lung function indicators (MD = 6.53, 95% CI: 1.03–12.03). Furthermore, family-centered nursing interventions significantly increased the symptom control rate (3 studies, RR = 5.24, 95% CI: 3.14–8.73) and caregiver knowledge mastery rate (3 studies, RR = 2.36, 95% CI: 1.30–4.29), while reducing the rehospitalization rate (3 studies, RR = 0.24, 95% CI: 0.11–0.55).

**Conclusion:**

Family-centered nursing intervention can effectively improve outcomes associated with clinical symptoms, lung function and quality of life in preschool children with asthma, enhance caregivers’ asthma management capacity and reduce the use of medical resources, which is worthy of popularization and application in clinical practice.

**Systematic Review Registration:**

https://inplasy.com/?s=INPLASY202670039, identifier INPLASY202670039.

## 1 Introduction

Asthma is one of the most common chronic respiratory diseases among children worldwide, with its incidence showing a continuous upward trend in the preschool child population (1). Multiple epidemiological surveys have shown that the global prevalence of asthma in children under 5 years old is about 4.8% (2, 3). Reported prevalence nevertheless differs markedly between regions: in the Global Asthma Network Phase I survey, the current prevalence of wheeze among children aged 6–7 years was 9.1% overall, ranging from approximately 5.6% in low-income and lower-middle-income countries of Asia and Africa to 11.6% in high-income countries of Europe and North America, with the highest regional estimate recorded in the Americas (approximately 16.3%) and the lowest in the South-East Asia and Western Pacific regions (approximately 7.8%) (4). Over the past 27 years, about one tenth of the global population still suffers from wheezing, and nearly half of them have severe symptoms, indicating a heavy disease burden (5). Asthmatic children often present with recurrent symptoms such as wheezing, coughing, and shortness of breath, which not only seriously affect their growth, development, and cognitive progress but also lead to increased rates of emergency department visits and hospitalizations, imposing a heavy medical burden on families and societies (6, 7). Children's daily life and health management are highly dependent on the care of family members. Studies have shown that the lack of family management ability is the primary factor leading to poor control of asthma in children (8, 9). Therefore, improving the family's asthma management ability is the key link to improve the prognosis of children (10). Family centered care(FCC) is a collaborative nursing model that centers around treating families as equal partners in the nursing process (11). FCC has demonstrated significant effectiveness in the management of chronic diseases such as those in intensive care units (12), pediatric diabetes (13), and congenital heart disease (14). However, its application effects in preschool children with asthma have not been systematically validated. Therefore, this study aims to integrate randomized controlled trials (RCTs) data on FCC in preschool children with asthma from both domestic and foreign sources through a systematic review and meta-analysis method, clarify its impact on the outcomes of children, help families improve management capabilities, reduce the risk of asthma attacks, improve the quality of life of children, and alleviate social burden. In China, asthma is also a common chronic respiratory disease in early childhood; a nationwide epidemiological survey of children in urban areas reported a cumulative asthma prevalence of about 3.0% among children under 14 years old, with the highest age-specific prevalence (4.15%) occurring in preschool children aged 3–5 years. Despite this, family-based asthma care in China still faces several practical pain points: parents frequently use inhalation devices (such as metered-dose inhalers with spacers and nebulizers) incorrectly, long-term adherence to inhaled controller medication is poor, peak-flow and nighttime blood-oxygen monitoring at home are often absent, and outpatient health education tends to be fragmented with insufficient home follow-up. In the same nationwide survey, inhaled corticosteroids were used by only 58.7% of asthmatic children while antibiotics were used by 75.1%, and peak-flow meters were used for monitoring by only 14.3% of children aged 5 years and older (15). These real-world clinical problems highlight the importance of building a structured, family-centered management model adapted to local conditions, and underscore the value of the present study in addressing native clinical needs.

## 2 Method

### 2.1 Research design

The present meta-analysis was conducted in accordance with the guidelines of the Preferred Reporting Items for Systematic Reviews and Meta-Analyses (PRISMA). Our meta-analysis protocol was registered in INPLASY (registration number: INPLASY202670039) and can be found at https://inplasy.com/inplasy-2026-7-0039/.

### 2.2 Literature retrieval strategy

A comprehensive search of both Chinese and English databases was conducted, including PubMed, Embase, Web of Science, CINAHL, and Scopus. A combination of subject headings and free-text terms was used for retrieval. The corresponding search terms are as follows: family-centered care intervention, preschool children, asthma, and randomized controlled trial. Taking PubMed as an example, the search equation is as follows: (family-centered care OR family-focused care) AND (preschool children OR 3–6 years old) AND asthma AND (randomized controlled trial OR RCT). The search timeframe spanned from the inception of each database to December 2025. Additionally, manual searches were conducted on the reference lists of included studies, and study authors were contacted to obtain unpublished or missing data.

### 2.3 Inclusion and exclusion criteria

Inclusion and exclusion criteria were developed according to the PICO (Population, Intervention, Comparison, Outcome) principle, as shown in Table 1.

**Table 1:** Inclusion and exclusion criteria.

Dimensions	Inclusion criteria	Exclusion criteria
Study type (T)	Randomized Controlled Trial (RCT), regardless of blinding status	Non-RCT studies (cohort study, case-control study, etc.), reviews, case reports, conference abstracts (without full text)
Study population (P)	3–6 years old preschool children; meeting the diagnostic criteria for asthma	Age <3 years or >6 years; comorbid with other chronic diseases (e.g., cystic fibrosis, congenital heart disease); severe cardiopulmonary insufficiency
Intervention (I)	Experimental group received family-centered care (FCC) intervention	Experimental group lacked core elements of FCC; intervention measures were unclear or showed no significant difference from routine care
Control (C)	Control group received routine care (e.g., outpatient health education, medication guidance, regular follow-up)	Control group received other novel interventions (e.g., Internet + nursing, multidisciplinary team intervention)
Outcome measures (O)	Included at least one preset outcome: 1. Disease control (asthma attack frequency, emergency/hospitalization rate, FEV1, C-ACT score) 2. Family management ability (asthma knowledge score, Morisky Medication Adherence Scale score, SEMS self-efficacy score)	No available outcome data; outcome measures irrelevant to preset ones; obvious data errors
Others	Full-text published; complete data	Duplicate publication; lack of ethical review

FCC, family-centered care; FEV1, forced expiratory volume in 1 s; C-ACT, Childhood Asthma Control Test; Morisky Medication Adherence Scale, a validated tool for assessing medication adherence; SEMS, Self-Efficacy for Managing Symptoms Scale.

### 2.4 Literature screening and data extraction

It was independently completed by two researchers, and studies that clearly did not meet the inclusion criteria were excluded based on their titles and abstracts. The full texts of studies that passed the initial screening were subsequently reviewed to determine their eligibility for inclusion. Any disagreements were resolved through discussion and consensus, with a third independent researcher consulted for arbitration when necessary. Using a standardized data extraction table, the extracted content includes: a) basic research information: author, publication year, country, sample size; b) Characteristics of subjects: age, sex ratio and asthma course; c) Intervention details: duration, frequency, implementation mode (outpatient/home) and core content of FCC intervention; d) Comparison measures: Specific content of routine care; e) Outcome measures: mean, standard deviation (SD), and sample size (n) of each indicator. After data extraction, a second researcher independently cross-checked the extracted information to ensure its accuracy and completeness.

### 2.5 Quality assessment of studies

The methodological quality and potential risk of bias in the included randomized controlled trials (RCTs) were assessed using the Cochrane risk-of-bias tool for randomized trials (RoB 2.0). The assessment encompassed the following five domains: bias arising from the randomization process; bias due to deviations from the intended interventions; bias due to missing outcome data; bias in the measurement of the outcome; and bias in the selection of the reported result. Each domain was rated as “low risk of bias,” “some concerns,” or “high risk of bias,” and an overall risk-of-bias judgement was then derived for each study: a study was judged to be at high overall risk of bias if at least one domain was rated as high risk or if multiple domains raised some concerns. The assessment was performed independently by two researchers, and any differences were resolved through discussion.

### 2.6 Data analysis

Meta-analysis was carried out using RevMan 5.4 software. a) Heterogeneity Assessment. Heterogeneity was assessed using the *I*^2^ statistic. *I*^2^ < 50% and *P* > 0.1 indicated acceptable heterogeneity, and a fixed-effect model was used. *I*^2^ ≥ 50% or *P* ≤ 0.1 indicated significant heterogeneity, and a random-effects model was employed. The sources of heterogeneity were further explored via subgroup or sensitivity analyses. b) Calculation of effect quantity. Continuous variables (such as asthma knowledge score and forced expiratory volume in 1 s [FEV1]): use weighted mean difference (WMD) or standardized mean difference (SMD), 95% confidence interval (CI); Categorical variables (such as emergency/hospitalization rate): use risk ratio (RR), 95% CI. c) subgroup analysis. According to the heterogeneous sources, the following subgroup analysis was carried out: intervention duration: ≤3 months vs. >3 months; implementation mode: outpatient intervention vs. home intervention; and study region: Asia vs. Africa vs. North America. d) Sensitivity Analysis. The robustness of the results was tested by: excluding low-quality studies (with ≥2 items rated as high risk of bias); excluding studies with a sample size <50; and switching the effect model. e) Publication Bias Assessment. If ≥10 studies were included, publication bias was assessed using funnel plots and Egger's test. If <10 studies were included, only a descriptive analysis was performed.

## 3 Results

### 3.1 Literature inclusion

#### 3.1.1 Literature retrieval and inclusion results

Through systematic search and screening, 15 randomized controlled trials were finally included, and the specific screening process is shown in Figure 1.

**Figure 1:**
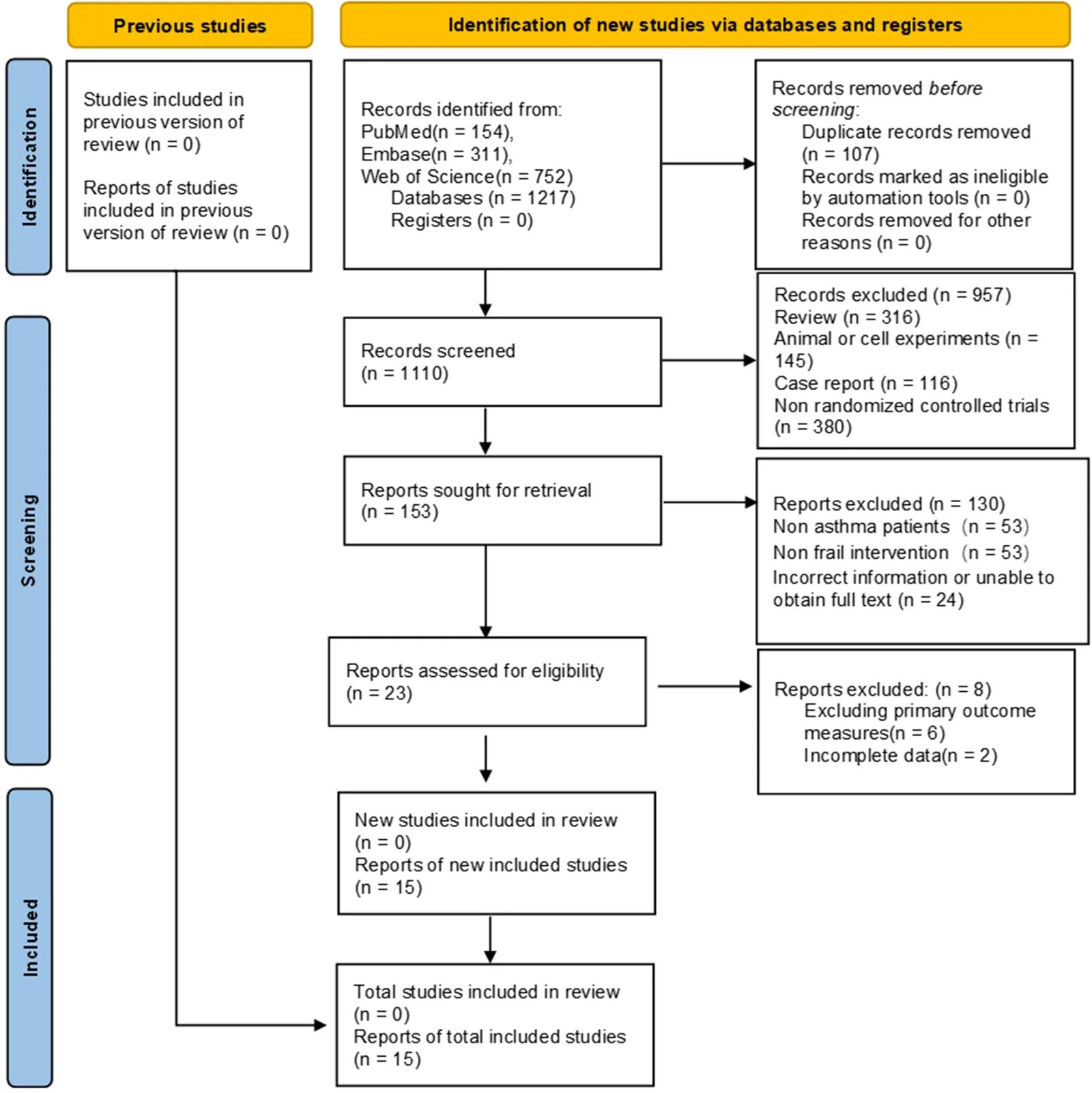
Study selection process.

#### 3.1.2 Basic characteristics of the included studies

A total of 15 randomized controlled trials (16–30) were included, involving six countries: Tunisia, Egypt, the United States, China, Iran, and Thailand. By region, three trials were conducted in Africa (Tunisia and Egypt), two in North America (the United States), and ten in Asia (China, including Hong Kong and Taiwan, Iran, and Thailand); no eligible trial was identified in Europe. The overall sample size consisted of 457 preschool children with asthma. The research subjects were preschool children with asthma aged 3–6 years, with an average age of 7.2 ± 2.5 years. The intervention group received family-centered nursing intervention, delivered through group discussion, home-based education, and telephone follow-up, and the intervention duration ranged from 2 weeks to 3 months. The control group received routine care. The follow-up period spanned from 2 to 12 months. Detailed information is presented in Table 2.

**Table 2:** Characteristics of included literatures.

Author, publication year	Country	Sample size of experimental group	Sample size of control group	Age	Gender ratio	Duration & frequency of FCC intervention	Follow-up
Dardouri et al. 2021 (16)	Tunisia	34	34	10.34 ± 2.35	32/36	Four group discussions, divided into 2 days, lasting 8 weeks	12 months
Dardouri et al. 2020 (17)	Tunisia	34	34	10.34 ± 2.35	32/36	Four educational sessions over 8 weeks (July–August 2018)	12 months
Eakin et al. 2020 (18)	USA	147	163	4.2[0.7]	159/151	Four 30–45 min home-based asthma management educational sessions + 3 intensive telephone follow-ups targeting preschoolers in urban family settings	12 months
Fouda et al. 2015 (19)	Egypt	23	24	8.91 ± 2.52	27/20	Twice weekly for 3 months	3 months
Gao et al. 2020 (20)	China	73	52	7.6 ± 2.1	64/61	Each session lasted ∼30 min; telephone consultations averaged 15 min each; total of four sessions	6 months
Jonas et al. 2022 (21)	USA	75	76	5.7 (2.0)	97/54	Six sessions, once every two weeks, each lasting one hour	12 months
Kashaninia et al. 2018 (22)	Iran	23	22	8.72 ± 1.56	28/17	–	6 months
Mahmoud et al. 2019 (23)	Iran	35	35	–	21/49	Five educational sessions, twice weekly, each lasting 45 min	3 months
Ng et al. 2021 (24)	China	56	56	5.26 (2.2)	82/30	45 min per session	6 months
Payrovee et al. 2014 (25)	Iran	14	16	7–11	17/13	Two 2-hour sessions weekly; two subgroups completed 4 intervention sessions over 4 weeks	3 months
Sangnimitchaikul et al. 2022 (26)	USA	37	37	6.0 ± 2.9	48/26	Three interactive meetings (60 min each) over two months	2 months
Shao et al. 2024 (27)	China	47	52	7.49 ± 1.18	56/43	–	3 months
Teymouri et al. 2017 (28)	Iran	30	30	10.16 ± 0.98	32/28	Seven 30-minute sessions	4 months
Wang et al. 2025 (29)	China	28	28	8.50 (2.01)	39/17	Five weeks	3 months
Yeh et al. 2016 (30)	China	34	31	9.23 ± 1.98	39/26	Four treatment sessions, each lasting 50 min	12 months

#### 3.1.3 Quality evaluation of included studies

According to the RoB 2.0 tool evaluation, 14 out of 15 studies had an overall “low-risk” bias risk, accounting for 93.33%. The specific distribution of bias risk is shown in Figure 2.

**Figure 2:**
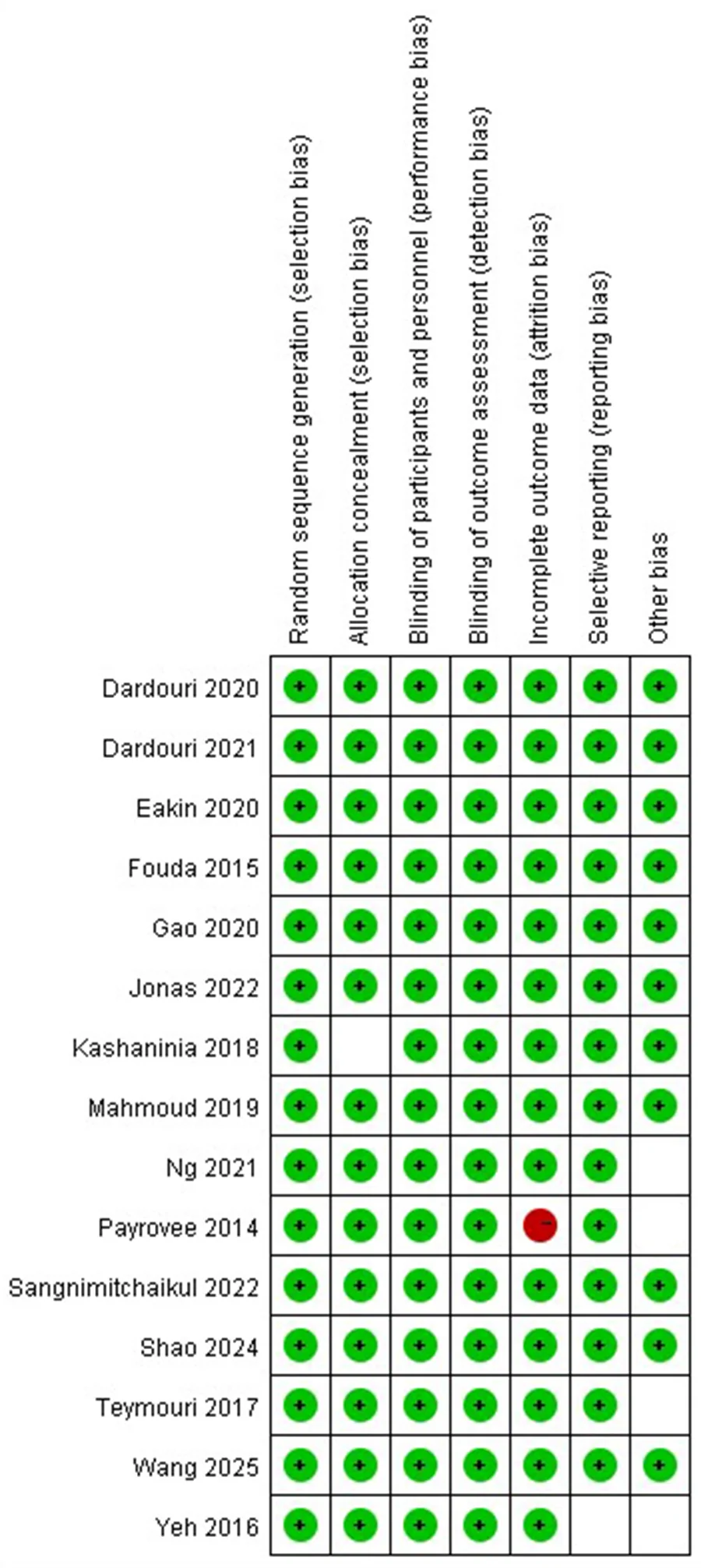
Results of literature quality evaluation.

### 3.2 Meta-analysis results

#### 3.2.1 C-ACT score

A total of 6 studies reported children's asthma control test (C-ACT) scores. Meta-analysis showed that *I*^2^ = 85%, and the pooled mean difference was 2.49 (95% CI: 1.15–3.83), *Z* = 3.63, *P* = 0.0003, indicating that the average C-ACT score of the experimental group was 2.49 (95% CI: 1.15–3.83) points higher than that of the control group. Further details are presented in Figure 3.

**Figure 3:**
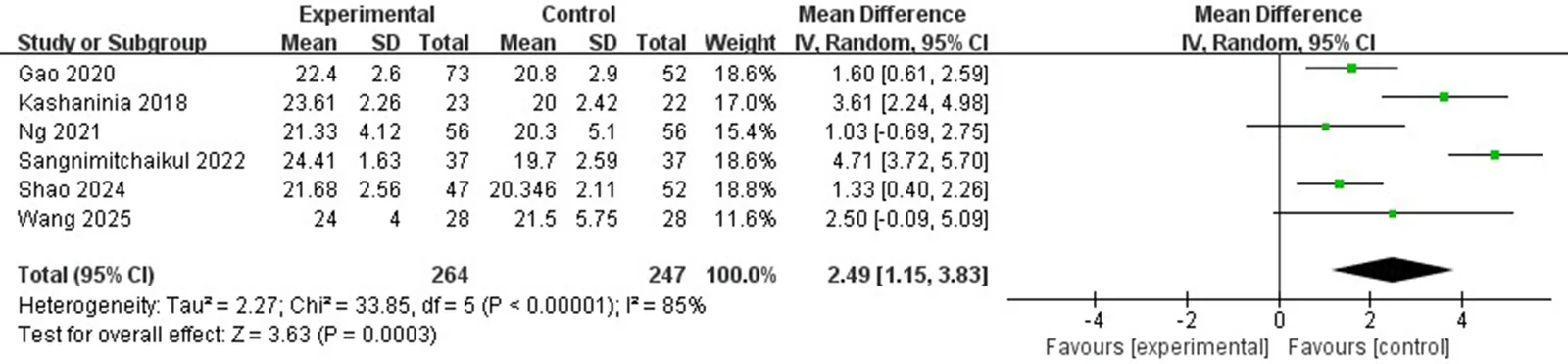
Forest plot of the impact of family-centered nursing interventions on C-ACT scores in children with asthma.

#### 3.2.2 Quality of life rating

Eight studies reported quality of life scores, with the Pediatric Asthma Quality of Life Questionnaire (PAQLQ) being the primary instrument; because other quality-of-life instruments were also used across the included studies, the standardized mean difference (SMD) was applied. Meta-analysis showed that *I*^2^ = 90%; a random-effects model was therefore selected, and the pooled standardized mean difference was 1.02 (95% CI: 0.52–1.51), *Z* = 4.00, *P* < 0.0001, indicating that the intervention group had significantly higher quality of life scores than the control group, and the difference was statistically significant. Further details are presented in Figure 4.

**Figure 4:**
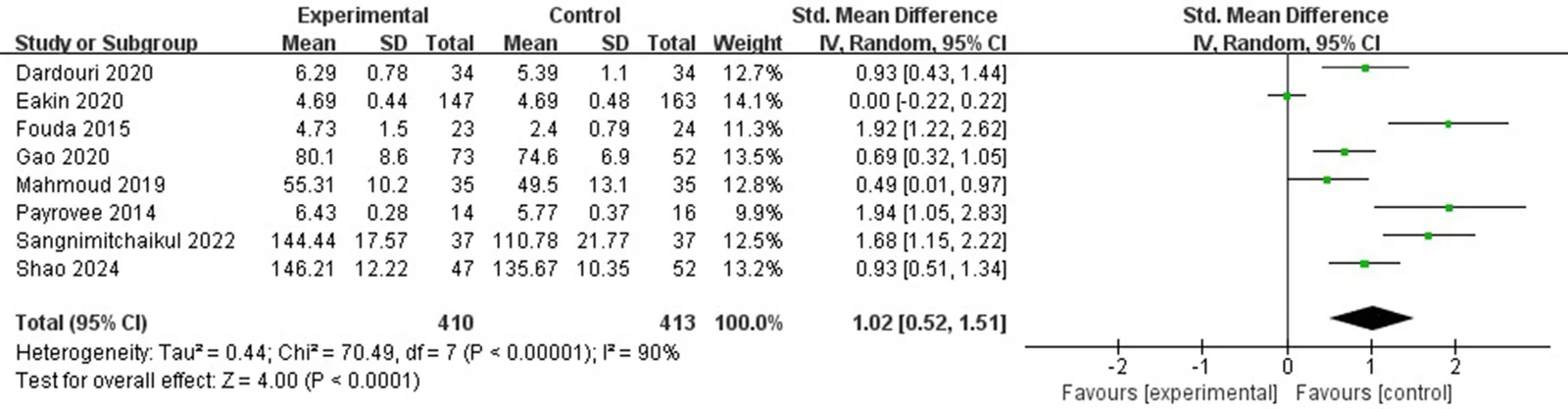
Forest plot of the impact of family-centered nursing interventions on quality of life scores in children with asthma.

#### 3.2.3 Lung function

Four studies reported the lung function index forced expiratory volume in 1 s/forced vital capacity (FEV1/FVC). Meta-analysis showed that *I*^2^ = 92%. A random-effects model was selected, and the pooled mean difference was 6.53 (95% CI: 1.03–12.03), *Z* = 2.33, *P* = 0.02, indicating that the pulmonary function of the intervention group was improved by 6.53% on average compared with the control group. Further details are presented in Figure 5.

**Figure 5:**
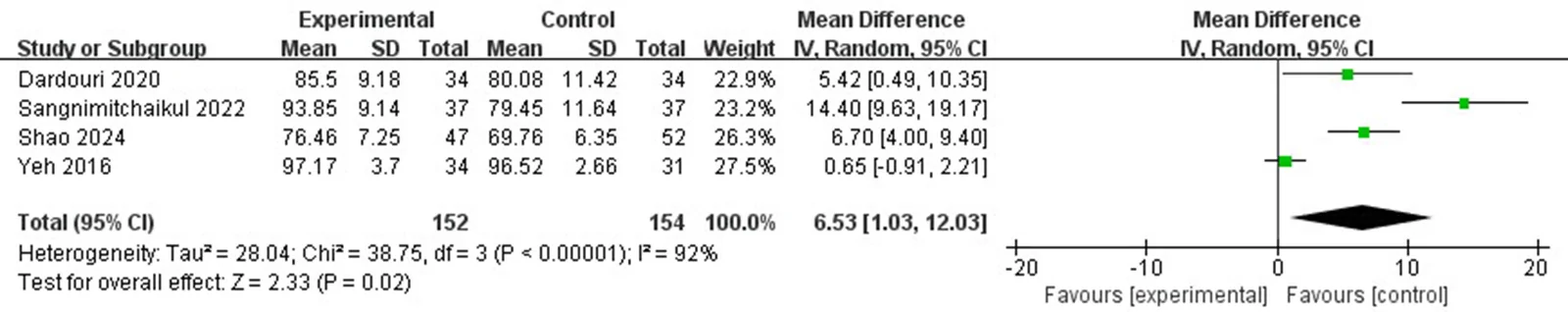
Forest plot of the impact of family-centered nursing interventions on lung function in children with asthma.

#### 3.2.4 Readmission rate

Three studies reported readmission rates. Meta analysis showed that family centered nursing interventions significantly reduced the risk of readmission in pediatric patients, with a risk ratio of 0.24 (95% CI: 0.11–0.55), indicating that the intervention group had a 76% reduction in readmission risk compared to the control group, and the difference was statistically significant (*Z* = 3.36, *P* = 0.0008). As shown in Figure 6.

**Figure 6:**
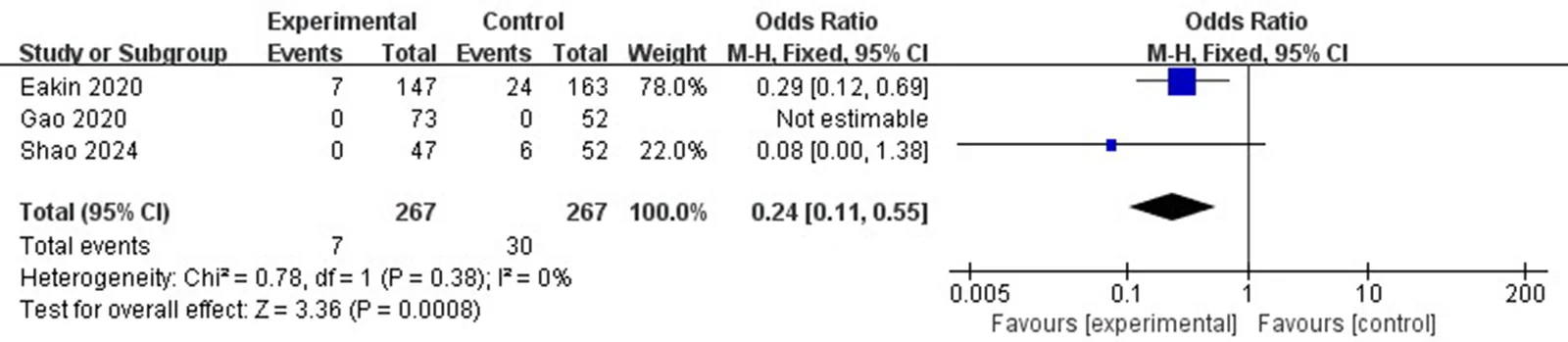
Forest plot of the impact of family-centered nursing interventions on readmission rates in children with asthma.

#### 3.2.5 Symptom control rate

Three studies reported symptom control rates. Meta-analysis showed that family-centered nursing intervention significantly improved the symptom control rate of children, and the risk ratio was 5.24 (95% CI: 3.14–8.73), the difference was statistically significant (*Z* = 6.35, *P* < 0.0001). As shown in Figure 7.

**Figure 7:**
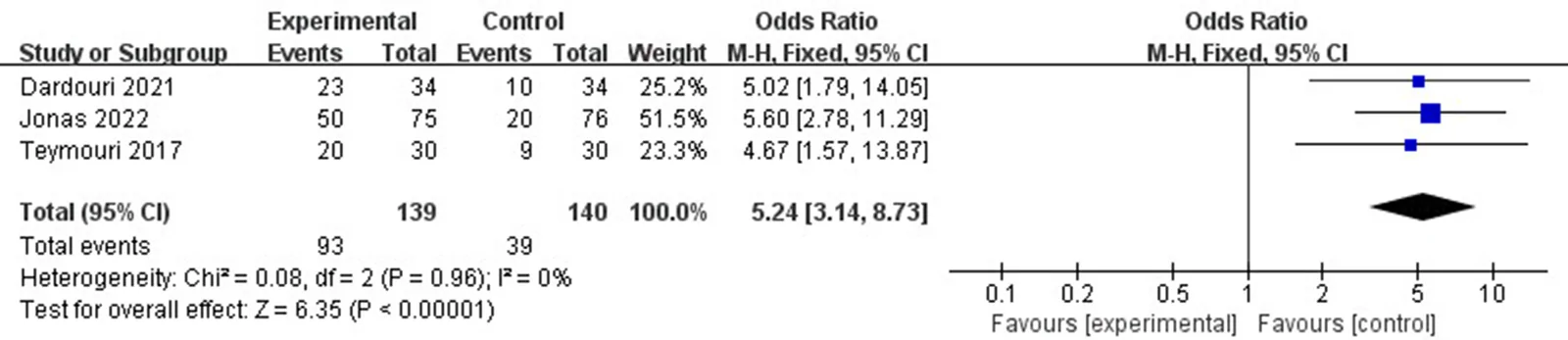
Forest plot of the impact of family-centered nursing interventions on symptom control in children with asthma.

#### 3.2.6 Knowledge mastery rate

Three studies reported knowledge mastery rates. Meta analysis showed that family centered nursing interventions significantly improved the knowledge mastery rate of pediatric patients, with a risk ratio of 2.36 (95% CI: 1.30–4.29), and the difference was statistically significant (*Z* = 2.81, *P* = 0.005). As shown in Figure 8.

**Figure 8:**
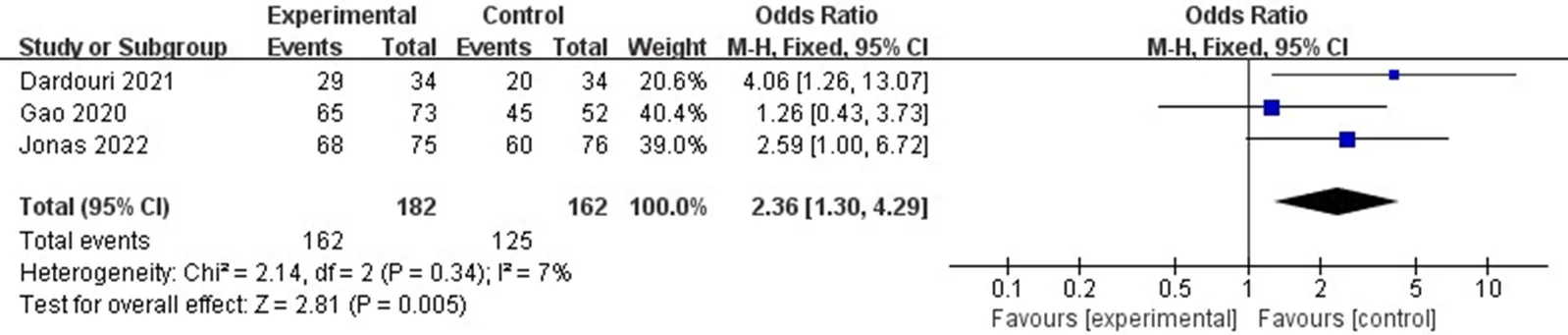
Forest plot of the impact of family-centered nursing interventions on caregiver knowledge mastery rates in children with asthma.

### 3.3 Sensitivity analysis

After excluding a “high-risk” study, the combined analysis was carried out again, and the direction and effect of the results of the quality of life score (SMD = 0.98, 95% CI: 0.48–1.48) did not change significantly, indicating that the results were more robust to the quality of the research methods. Upon excluding three small-sample studies (total sample size < 60), the effect size for lung function improvement slightly decreased (MD = 5.87, 95% CI: 0.95–10.79) but remained statistically significant, suggesting that small-sample studies may have somewhat overestimated the intervention effect. The sensitivity analysis results showed that, except for lung function indicators being more sensitive to small sample studies, the other main conclusions did not undergo substantial changes, indicating that the overall meta-analysis results of this study are robust.

### 3.4 Publication bias

As the number of studies included for each outcome indicator was less than 10, which does not meet the minimum recommended number of studies for conducting funnel plot symmetry tests or Egger’s test, a formal statistical test for publication bias was not performed.

## 4 Discussion

This systematic review and meta-analysis included 15 randomized controlled trials, involving 457 preschool children with asthma. The results showed that family-centered care (FCC) could significantly improve children's asthma symptom control, lung function and quality of life, and effectively enhance the family's ability to master and manage asthma-related knowledge and reduce the risk of re-hospitalization. Overall, FCC, as a nursing model that emphasizes family participation, collaborative decision-making, and ongoing education, has good clinical value in the management of preschool asthma.

The results of this study indicate that FCC intervention can significantly improve children's C-ACT scores and symptom control rates while reducing readmission rates, suggesting its definite efficacy in asthma control. FCC interventions typically include family education, personalized counseling, home guidance, and follow-up management (31, 32). FCC intervention can improve parents’ mastery of inducement identification, inhalation device use and acute attack treatment strategy. Enable families to make scientific judgments in the daily lives of sick children, reducing treatment delays or improper management, thereby improving symptom control in children (33, 34). In addition, this study found that FCC intervention can significantly improve the FEV1/FVC index of children, indicating that it also plays a positive role in promoting the improvement of airway function. Possible mechanisms include family mastery of correct inhalation therapy methods, adherence to standardized long-term control medication (such as inhaled corticosteroids, ICS) treatment, and environmental management behaviors that reduce exposure to allergens or triggering factors (35). This aligns with previous studies concluding that family education interventions can enhance lung function and control levels in preschool children with asthma (36, 37), further supporting the effectiveness of the FCC model in managing chronic respiratory diseases in children. These mechanisms are highly consistent with the core principles of current asthma management guidelines. Both the Global Initiative for Asthma (GINA) and the 2024 edition of the Chinese guideline for the diagnosis and prevention of childhood asthma emphasize that asthma is essentially a chronic airway inflammatory disease, and that sustained control depends on regular maintenance therapy with ICS, correct inhalation technique, and systematic allergen and trigger avoidance. Within the FCC model, parents act as the primary executors of home management, and by mastering ICS maintenance, standardized device operation, and environmental control, they directly support the anti-inflammatory and airway-protective goals advocated by these guidelines, thereby linking improved family management capacity to the suppression of chronic airway inflammation and the improvement of lung function (45). Nevertheless, the available evidence is not uniformly positive. A cluster-randomized trial of a comprehensive telemedicine-delivered asthma education programme reported no significant between-group difference in symptom-free days, quality of life, self-efficacy, asthma knowledge, or lung function, despite improved peak-flow monitoring and self-reported adherence (38); likewise, a meta-analysis of paediatric asthma education found a reduction in emergency department visits but no effect on the odds of hospitalization (39). These divergent findings suggest that the benefit of family-centred approaches is strongly dependent on intervention intensity, delivery format, and baseline family resources, and that the pooled estimates reported here should be interpreted with corresponding caution.

The core management responsibility for preschool asthma lies with the family, and parents’ disease knowledge, behavioral habits, and health beliefs directly affect the effectiveness of disease control in children (40, 41). This study demonstrates that FCC can significantly improve families’ asthma knowledge mastery and self-management capabilities, which is highly consistent with FCC's emphasis on the family's leading role in disease management (42). Comparable evidence from other chronic conditions supports this principle: a recent systematic review of family involvement in schizophrenia care concluded that family-centred care depends on open knowledge exchange, mutual respect, and the empowerment of relatives to share in treatment decisions (43). The FCC model strengthens parents’ active learning and problem feedback through multi-dimensional interventions, such as group discussion, family consultation, scenario simulation, telephone follow-up, etc., so that they can more skillfully cope with children's symptom changes, medication management and environmental control needs (44). Simultaneously, FCC establishes bidirectional communication between the family and the healthcare team, allowing parents to continuously receive professional support during the intervention period, thereby increasing their self-efficacy and promoting long-term behavioral changes (45). Existing research indicates that improved family management capacity can significantly reduce the risk of future acute asthma attacks in children (46). In the meta-results of this study, the pooled readmission rate in the FCC group was reduced by 76%, which further confirmed the important role of strengthening family management ability in improving long-term prognosis. In real-world clinical practice, the absence of competent home care is a direct trigger for poor outcomes: when parents fail to recognize early warning signs, misuse inhalation devices, or do not maintain controller medication, preschool children are prone to recurrent wheezing, nighttime declines in blood oxygen saturation, and acute exacerbations that culminate in emergency-department visits and re-hospitalization. These clinical realities echo the quality-control problems frequently identified in pediatric respiratory wards, such as incomplete documentation of symptom diaries and the lack of structured home monitoring records, which reflect a disconnect between hospital-based care and continuous family management. Compared with the relatively mature structured FCC programs reported abroad, current pediatric respiratory nursing in China still relies heavily on fragmented outpatient education, with insufficient home follow-up and frequently non-standardized parental operation of nebulizers and peak-flow meters. The FCC model, by embedding assessment, education, follow-up, and feedback into a closed loop, offers a practical pathway to bridge this gap. Accordingly, FCC implementation can be tailored to distinct care scenarios: in the respiratory ward, nurses can deliver structured bedside education and device-operation training before discharge; in the outpatient clinic, individualized counseling and trigger-identification guidance can be reinforced at each visit; and in home-based continuity care, telephone or online follow-up, home nebulization management, and home blood-oxygen monitoring can be combined to sustain control between visits.

Quality of life is a crucial outcome in asthma management. This study found that Family-Centered Care (FCC) interventions can significantly improve the comprehensive quality of life scores of children with asthma. The possible reasons include a reduction in the frequency of symptoms, decreased interference from acute exacerbations, improved sleep quality, and a reduction in limitations on children's physical activity and daily routines (47). At the same time, the improvement of family's emotional support, nursing participation and communication style can make children obtain a more stable psychological environment and help improve their overall quality of life (48). FCC focuses on establishing partnerships with family members to help parents better understand the emotional needs and behavioral responses of children with chronic diseases. This has a promoting effect on improving the emotional experience and social activity participation of children with chronic diseases, which is consistent with previous research on FCC improving the quality of life of children with chronic diseases (49).

There was high heterogeneity in some outcome indicators in this study, which may stem from the following sources: a) Significant variations in FCC intervention content: The studies differed in terms of intervention frequency, duration, and implementation methods (in-person instruction, home-based guidance, telephone follow-ups). b) The study area and cultural background are different: differences in family education methods, health concepts and medical resources may affect the intervention effect. c) The severity of asthma is inconsistent: some studies did not analyze the asthma in strict accordance with the classification. d) Different follow-up durations: There may be differences in the evaluation results of symptom control and quality of life between short-term and long-term follow-up. Although high heterogeneity may affect the stability of some effects, sensitivity analysis shows that the main conclusions are in the same direction, suggesting that the overall results are highly reliable. The results of this study support FCC as an important strategy for family management of preschool children with asthma, which should be further promoted in clinical nursing work. a) Establish a standardized FCC nursing process, including assessment, education, follow-up, and feedback mechanisms. b) Strengthen parent training to enhance their ability to identify asthma triggers and manage acute exacerbations. C) Promote multi-channel intervention mode (outpatient education + home guidance + telephone follow-up) to ensure the continuity of intervention. d) Promote the establishment of medical staff family partnership, reduce communication barriers, and improve nursing compliance. In future practice, digital health tools (such as mobile apps and self-monitoring systems) can be integrated into the FCC model to further enhance intervention accessibility and long-term management effectiveness (50). For preschool children specifically, several targeted, clinically actionable nursing recommendations can be embedded in the FCC framework. First, for home nebulization management, nurses should train parents in correct drug preparation, mask fitting, breathing coordination, and device cleaning and disinfection, and should provide a simple checklist so that each home session is standardized. Second, for home blood-oxygen monitoring, families of children with moderate-to-severe asthma or frequent nocturnal symptoms can be guided to use a pediatric pulse oximeter, to record daytime and nighttime SpO2 values, and to understand the threshold values that warrant medical attention. Third, for family first aid during acute attacks, parents should be taught a stepwise home action plan, including recognition of severe-attack signs, correct use of a reliever inhaler with a spacer, positioning and reassurance of the child, and clear criteria for seeking emergency care. These measures convert the abstract FCC model into concrete bedside and home-care nursing tasks that frontline pediatric respiratory nurses can implement. Furthermore, aligning with the current focus on smart management of pediatric chronic respiratory disease, the FCC model can be digitally upgraded: home blood-oxygen monitoring devices can transmit data to the care team for early warning, online parent classes can deliver standardized and repeatable education, and a nebulization follow-up mini-program can be used to push medication reminders, collect symptom diaries, and schedule remote follow-up. Such a digitally enhanced FCC pathway can improve the timeliness, traceability, and continuity of home asthma management for preschool children.

This study still has the following limitations: a) Inconsistent intervention methods: As a comprehensive care model, Family-Centered Care (FCC) has diverse specific implementation contents, which may lead to differences in intervention effects. b) Limited follow-up duration: The follow-up periods of the included studies ranged widely from only 2 to 12 months, and none provided long-term outcome data beyond one year. As a result, the long-term effect of FCC on children's distal lung-function development and on sustained asthma remission could not be evaluated, and whether the observed short-term benefits can be maintained over the long term remains to be confirmed. c) Incomplete report of some research data: information such as intervention details and asthma severity classification is missing, which affects the quality of comprehensive analysis. d) There is potential publication bias: the small sample size may affect the stability of the results. Suggestions for future research focus on: a) Unify FCC intervention contents and establish a standardized intervention framework; b) Conduct large-scale, multi center randomized controlled trials (RCTs); c) Extend follow-up time and evaluate the impact of FCC on long-term control and lung function development; d) Explore the differences of intervention under different family backgrounds (education level and economic status); e) Explore the mixed family management mode with information tools.

### 4.1 Strengths and implications for practice

This review has several strengths. To the best of our knowledge, it is the first systematic review and meta-analysis to focus specifically on family-centered nursing interventions in preschool children with asthma, an age group in which day-to-day disease management depends almost entirely on caregivers. The protocol was registered prospectively in INPLASY, the review was reported in accordance with PRISMA, only randomized controlled trials were eligible, and risk of bias was appraised with RoB 2.0, with 93.33% of the included trials judged to be at overall low risk of bias. A broad and clinically meaningful set of outcomes was synthesized, spanning symptom control, lung function, quality of life, caregiver knowledge, and use of health services, and the robustness of the pooled estimates was supported by sensitivity analyses. For practice, these findings indicate that family-centered care should be embedded in routine pediatric asthma services rather than delivered as *ad hoc* education. Nursing teams can operationalize it through structured pre-discharge device training, individualized trigger-identification counselling at each outpatient visit, and scheduled telephone or online follow-up that closes the loop between hospital and home. Because such nurse-led family partnership programmes require no additional pharmacological resources, they are feasible in both well-resourced and resource-limited pediatric settings, and they offer a practical route to reducing avoidable exacerbations and readmissions in preschool children with asthma.

## 5 Conclusion

Family-centered care intervention can significantly improve disease control, lung function, and quality of life in preschool children with asthma, enhance family disease management capabilities, and reduce the use of medical resources. This study provides evidence support for the promotion of FCC in childhood asthma management, emphasizing the importance of family participation in nursing practice and the need to build a collaborative care model to achieve better health outcomes. Accordingly, family-centered care interventions should be considered for integration into routine asthma management for preschool children.

## Data Availability

The original contributions presented in the study are included in the article/Supplementary Material, further inquiries can be directed to the corresponding author.
